# Comparison of vision-related quality of life in patients with homonymous hemianopia and monocular blindness

**DOI:** 10.1038/s41598-022-10626-w

**Published:** 2022-04-21

**Authors:** Hee-young Choi, Su-Jin Kim, Sang Yoon Kim, Ji-Eun Lee

**Affiliations:** 1 Department of Ophthalmology, Pusan National University KR, Biomedical Research Institute Pusan National University Hospital, Busan, Korea; 2 Department of Ophthalmology, Pusan National University School of Medicine and Research Institute for Convergence of Biomedical Science and Technology, Pusan National University Yangsan Hospital, 20-Geumo-ro, Mulgeum-eup, Yangsan, Gyeongsangnam-do 50612 South Korea; 3 Department of Ophthalmology, Stanford University School of Medicine, Palo Alto, CA USA

**Keywords:** Quality of life, Stroke, Eye manifestations

## Abstract

To evaluate the vision-related quality of life (QoL) in patients with homonymous hemianopia (HH). The study compared the QoL in 32 patients with HH and 33 patients with monocular blindness. Best-corrected visual acuity (BCVA) and visual field test were investigated. The National Eye Institute-Visual Function Questionnaire 25 (NEI VFQ-25) and independent mobility questionnaires (IMQs) were used to assess their perceived visual and physical functioning abilities. The results of QoL questionnaires were compared in two groups. The mean deviation (MD) in the better eye was significantly lower in the HH group than in the monocular blindness group. The composite scores of NEI-VFQ and IMQs were significantly lower in the HH patients than in the monocular blindness patients. The driving-related score was significantly lower in patients with right hemianopsia than in those with left hemianopsia. The outdoor activity-related score was significantly lower in patients aged less than 55 years than in patients aged 55 years and more. Homonymous hemianopia had a negative impact on patients’ QoL by limiting their vision related activities compared to monocular blindness. The MD of the better eye in the HH patients reflects the binocular visual field and can affect the real visual function and QoL.

## Introduction

Homonymous hemianopia(HH) is the loss of half the visual field in both eyes on the same side and to the same extent. The visual field loss results from post-chiasmal damage to the optic tract or its cortical projections and is contralateral to the side of the brain injury^1^. Stroke is the most prevalent cause of HH, followed by severe brain injury, malignancies, and brain surgery^2^. Aging is the most robust non-modifiable risk factor for incident stroke, which doubles every 10 years after age 55 years. As the number of people aged ≥ 65 years is projected to grow, the number of strokes in older adults is expected to rise^3^. And a decrease in stroke mortality increases the number of stroke survivors living with residual impairment^4,5^, including visual field defects (VFDs). The prevalence of visual field loss following a stroke has been to be as high as 28–63% in acute stroke units^6,7^. Many of these patients do not regain their lost visual field. The impact on affected individuals can be substantial. This has far-reaching and profound consequences for patients’ psychological, vocational and personal lives^8,9^.

Understanding how HH affects daily functioning and quality of life (QoL) can help improve clinical assessment and rehabilitation strategies. Some studies have reported that HH can have a negative impact on patients' general and vision-related QoL using self-reported questionnaires^10–14^. Furthermore, the association between visual impairment (VI) and disability inactivities of daily living has been well-established, indicating that the severity of VI worsens overall QoL^15,16^.

To the best of our knowledge, none of the studies compared the difficulties experienced by patients with HH with those experiencing monocular blindness, although reports have described QoL in patients compared with healthy individuals with normal visual field. Hence, this study compared the QoL between patients with HH and those with monocular blindness.

## Results

### Baseline characteristics

Between March 2019 and February 2021, 65 patients were enrolled at a single site. The average age was 53.9 ± 16.0 years, and 36 (55.4%) subjects were male. Table 1 lists the baseline characteristics of each group. The MD in better eye was significantly lower in the HH group than in the monocular blindness group. BCVA in worse eye was significantly better in the HH group. No differences in other parameters were found between the two groups.

**Table 1:** Patient demographics in two groups.

	Homonymous hemianopia (n = 32)	Monocular blindness (n = 33)	*p*-value
Sex (male/female)	19:13	17:16	0.62
Age, mean ± SD (years)	53.0 ± 13.90	54.7 ± 13.9	0.28
Time from symptom onset to enrollment median(range), (months)	11.2 (6–25)	9.6 (5–20)	0.45
**Best corrected visual acuity, mean ± SD (logMAR)**			
Better eye	0.04 ± 0.06	0.04 ± 0.05	0.15
Worse eye	0.08 ± 0.09	2.4 ± 0.71	< 0.0001
**Mean deviation, mean ± SD (dB)**			
Better eye	− 14.51 ± 3.76	− 1.96 ± 0.37	< 0.0001
Worse eye	− 18.70 ± 5.08	Uncheckable	

### Patients demographics

Thirty-seven patients referred by neuro-ophthamology clinics were screened for participation. Of these, 32 (19 male) patients who were enrolled to the HH group had an average age of 53.0 ± 13.97 years (range 24–73 years). Based on the etiology of hemianopia, stroke was the cause in 22 patients, brain tumor resection in 7 cases, and traumatic brain injury in 3 subjects. At time of study enrollment, the median time from injury was 11.2 (range 6–25) months. The median binocular visual acuity of enrolled patients was − 0.10 logMAR (20/16, range 20/11–20/32). Seventeen patients had right hemianopia and 15 had left hemianopia.

Thirty-three patients were assigned to the monocular blindness group. The average age was 54.7 ± 13.93 years (range 43–77). The etiology of unilateral visual loss included non-arteritic anterior optic neuropathy (13 cases), retinal detachment (6 cases), age-related macular degeneration (10 cases) and traumatic optic neuropathy (4 cases). The median time from symptom onset to enrollment was 9.6 (range 5–20) months (Table 1).

### Primary outcome

The National Eye Institute Visual Function Questionnaire (NEI VFQ-25) scores of general health, near activities, distance activities, social functioning, mental health, role difficulties, dependency, driving, peripheral vision and color vision were significantly lower in the HH group than in the monocular blindness group. However, the scores of general vision and ocular pain were not significantly different between the two groups (Table 2). Furthermore, all of the independent mobility questionnaires (IMQs) scores except Q24, 29, 30 were significantly lower in the HH group than in the monocular blindness group (Table 3).

**Table 2:** Analysis of National Eye Institute-Visual Function Questionnaire (NEI-VFQ) between homonynous hemianopia and monocular blindness group.

Subdomain	Questionnaires	Homonymous hemianopia	Monocular blindness	*p*-value	Right Hemianopia	Left hemianopsia	*p*-value
General health	1	46.5 ± 21.4	65.0 ± 27.6	**0.04**	42.6 ± 20.8	49.9 ± 23.5	0.46
General vision	2	38.2 ± 23.6	55.0 ± 28.4	0.06	37.7 ± 31.8	39.5 ± 13.7	0.45
Ocular pain	4, 19	74.2 ± 24.2	66.7 ± 30.0	0.58	72.0 ± 22.2	77.1 ± 25.1	0.46
Near activities	5, 6, 7	34.9 ± 24.1	58.9 ± 28.5	**< 0.01**	35.8 ± 20.7	33.9 ± 24.5	0.38
Distance activities	8, 9, 14	29.8 ± 21.2	59.9 ± 30.2	**< 0.01**	30.5 ± 20.3	30.4 ± 24.4	0.31
Social functioning	11, 13	39.7 ± 26.1	71.0 ± 31.7	**< 0.01**	40.3 ± 25.3	39.4 ± 26.3	0.44
Mental health	3, 21, 22, 25	41.7 ± 24.6	68.7 ± 28.8	**< 0.01**	40.8 ± 24.7	43.0 ± 28.5	0.38
Role difficulties	17, 18	35.7 ± 24.2	62.9 ± 28.7	**< 0.01**	34.0 ± 20.4	38.0 ± 30.6	0.45
Dependency	20, 23, 24	46.1 ± 27.4	70.8 ± 34.8	**< 0.01**	44.6 ± 27.4	48.1 ± 31.1	0.40
Driving	15c, 16, 16a	32.5 ± 27.5	64.6 ± 26.4	**0.02**	15.7 ± 16.1	51.4 ± 33.8	**0.04**
Peripheral vision	10	28.8 ± 18.6	57.5 ± 23.7	**< 0.01**	29.8 ± 15.0	24.4 ± 17.3	0.22
Color vision	12	52.3 ± 24.6	71.0 ± 20.8	**< 0.01**	49.5 ± 23.3	55.1 ± 26.9	0.38

**Table 3:** Analysis of independent mobility questionnaire (IMQ) between homonymous hemianopia and monocular blindness group.

Independent mobility questionnaires	Homonymous hemianopia	Monocular blindness	*p*-value	Right hemianopia	Left hemianopia	*p*-value
1. Walking in familiar areas?	3.0 ± 1.4	4.4 ± 0.7	**< 0.01**	3.1 ± 1.1	2.7 ± 1.8	0.36
2. Walking in unfamiliar areas	2.1 ± 0.9	3.4 ± 1.6	**< 0.01**	2.0 ± 0.7	2.1 ± 1.2	0.39
3. Moving about at home	3.2 ± 1.5	4.5 ± 0.9	**0.02**	3.4 ± 1.2	2.8 ± 1.6	0.35
4. Moving about at work?	2.5 ± 1.4	4.0 ± 1.4	**< 0.01**	2.7 ± 1.5	2.0 ± 1.3	0.17
6. Moving about stores?	2.4 ± 1.1	4.1 ± 0.9	**< 0.01**	2.4 ± 1.0	2.1 ± 1.2	0.30
7. Moving about outdoors?	2.5 ± 1.2	4.1 ± 1.6	**< 0.01**	2.9 ± 1.4	2.1 ± 1.2	0.14
8. Moving about in crowded situations?	1.8 ± 0.7	3.4 ± 1.8	**< 0.01**	1.9 ± 0.6	1.7 ± 0.8	0.31
9. Walking at night	1.9 ± 0.8	3.0 ± 1.5	**0.04**	2.0 ± 0.9	1.8 ± 0.7	0.30
10. Moving about using public transportation?	2.4 ± 1.3	3.8 ± 1.3	**< 0.01**	2.4 ± 1.1	2.1 ± 1.3	0.32
13. Walking up steps?	2.5 ± 0.9	3.6 ± 1.6	**0.01**	2.7 ± 0.9	2.4 ± 1.1	0.32
14. Walking down steps?	2.6 ± 1.1	3.5 ± 1.8	**0.04**	2.6 ± 0.7	2.9 ± 1.1	0.40
15. Stepping onto curbs?	2.6 ± 1.0	3.9 ± 1.5	**< 0.01**	2.8 ± 1.0	2.3 ± 1.1	0.18
16. Stepping off curbs?	2.4 ± 1.0	3.9 ± 1.7	**< 0.01**	2.4 ± 0.6	2.1 ± 1.1	0.30
17. Walking through doorways?	2.8 ± 1.3	3.9 ± 1.4	**0.02**	2.9 ± 1.3	2.3 ± 1.4	0.19
23. Walking in dimly light indoor areas	2.5 ± 1.5	4.0 ± 1.6	**0.01**	2.2 ± 1.3	2.8 ± 1.7	0.20
24. Being aware of another person’s presence?	2.8 ± 1.2	3.6 ± 1.4	0.05	2.8 ± 1.4	2.4 ± 1.0	0.29
25. Avoiding bumping into people?	2.4 ± 1.3	3.4 ± 1.2	**0.02**	2.6 ± 1.4	1.7 ± 0.8	0.09
26. Avoiding bumping into walls?	2.4 ± 1.2	3.3 ± 1.3	**0.03**	2.7 ± 1.5	1.7 ± 0.8	0.07
27. Avoiding bumping into head height objects?	2.5 ± 1.2	3.7 ± 1.2	**< 0.01**	2.8 ± 1.4	2.0 ± 1.2	0.13
28. Avoiding bumping into shoulder height objects?	2.5 ± 1.2	3.7 ± 1.3	**< 0.01**	2.7 ± 1.4	1.9 ± 0.7	0.11
29. Avoiding bumping into waist height objects?	2.6 ± 1.4	3.2 ± 1.3	0.05	2.8 ± 1.6	2.4 ± 1.2	0.21
30. Avoiding bumping into knee height objects?	2.7 ± 1.4	3.4 ± 1.4	0.09	2.8 ± 1.4	2.7 ± 1.7	0.46
31. Avoiding bumping into low lying objects?	2.5 ± 0.9	3.7 ± 1.2	**< 0.01**	2.7 ± 0.9	2.3 ± 1.1	0.23
32. Avoiding tripping over uneven travel surfaces?	2.1 ± 0.6	3.3 ± 1.4	**< 0.01**	2.1 ± 0.6	1.9 ± 1.2	0.22
33. Moving around in social gatherings	2.4 ± 1.8	3.6 ± 1.2	**< 0.01**	2.4 ± 1.0	1.9 ± 1.2	0.11
35. Seeing cars at intersections?	2.5 ± 1.1	3.5 ± 1.3	**0.01**	2.6 ± 1.0	2.0 ± 1.0	0.15

### Secondary outcome

In the HH group, the score of subdomain involving driving in NEI-VFQ were significantly lower for the right hemianopsia than in the left hemianopsia (*p* = 0.04). Responses to other questionnaires were not significantly different between patients with right and left hemianopsia (Table 2). All of the IMQ scores was not significantly different between right and left hemianopsia (Table 3). The scores for each subdomains were not significantly different between male and female. Patients under the age of 55 had a greater negative impact on distance activities than patients aged 55 and over (*p* = 0.03) (Table 4).

**Table 4:** Comparison of National Eye Institute-Visual Function Questionnaire (NEI-VFQ) according to the age and sex.

Subdomain	Male	Female	*p*-value	age > 55	age ≤ 55	*p*-value
General health	44.2 ± 25.2	48.8 ± 27.6	0.24	44.7 ± 24.4	48.3 ± 25.9	0.27
General vision	32.5 ± 17.7	42.9 ± 27.8	0.17	43.2 ± 25.2	32.2 ± 18.8	0.13
Ocular pain	75.4 ± 25.5	73.0 ± 22.9	0.26	70.5 ± 29.5	77.9 ± 31.4	0.29
Near activities	25.0 ± 22.4	42.9 ± 27.8	0.08	39.2 ± 19.1	30.6 ± 33.6	0.21
Distance activities	22.3 ± 20.8	35.4 ± 12.2	0.10	41.7 ± 15.8	17.9 ± 12.2	**0.03**
Social functioning	37.2 ± 23.7	42.2 ± 28.5	0.16	36.8 ± 22.4	42.6 ± 27.6	0.15
Mental health	43.2 ± 19.7	53.6 ± 30.4	0.19	40.2 ± 22.5	43.2 ± 26.7	0.49
Role difficulties	32.1 ± 22.4	39.3 ± 31.8	0.14	29.9 ± 19.0	38.1 ± 37.4	0.28
Dependency	38.3 ± 27.2	45.1 ± 28.6	0.31	45.2 ± 24.6	47.0 ± 32.4	0.46
Driving	41.7 ± 35.4	23.3 ± 18.6	0.07	30.7 ± 37.8	35.0 ± 37.9	0.43
Peripheral vision	27.5 ± 17.5	29.9 ± 14.4	0.29	36.2 ± 18.8	21.4 ± 17.3	0.10
Color vision	49.2 ± 23.2	55.4 ± 26.0	0.30	50.1 ± 25.6	54.5 ± 28.4	0.27

## Discussion

QoL has been analyzed in different ophthalmologic diseases associated with visual field (VF) loss such as glaucoma, retinal disorders, and optic neuropathy^17–19^. Many studies have found that patients with HH experience impaired general and vision-related QoL^10–12,20–22^. Eleni et al. found that patients with HH related to cerebrovascular disease scored lower on all subscales except ocular pain when using the VFQ-25 to assess QoL, suggesting that patient’s vision-targeted QoL as well as general health and composite scores were significantly worse than in healthy controls^11^. It is not surprising that HH is apparently correlated with a general deterioration in perceived visual function compared with heathy controls.

A recent meta-analysis reported that crude global prevalence of avoidable vision impairment and blindness in adults aged 50 years and older did not change between 2010 and 2019. Although, age-standardized prevalence of avoidable blindness decreased by 15.4%, the number of cases increased for both avoidable blindness and visual impairments^23^. Low vision (LV) has a considerable impact on QOL, indicating that the severity of visual impairment worsens overall QoL^15,16^. A meta-analysis of studies evaluating the QoL of LV patients reported that LV was associated with poor QoL and higher odds of depressive symptoms compared with healthy controls^24^.

This study differs from previous studies in that we compared the QoL of patients with HH with those with monocular blindness. In this study, the NEI-VFQ scores of patients with HH on each scale were significantly lower than in patients with monocular blindness except in terms of general vision and ocular pain. Furthermore, the QoL scores regarding mobility of patients with HH were significantly lower than in patients with monocular blindness. It is noteworthy that the perceived QoL of patients with HH was significantly lower than that of patients with monocular blindness. The loss of homonymous hemianopic visual field may have larger negative impact on patients’ QoL, including concerns about general health, outdoor activities, psychosocial distress, role difficulty and driving.

Visual field loss can make it difficult for people to function normally—especially moving about freely, avoiding obstacles, reading, driving, and taking part in rehabilitation for other stroke-related problems. Studie have demonstrated that people with visual field defects have an increased risk of accidents and falling^25^. People report walking into objects, tripping and falling, feeling unsafe, getting lost, and experiencing panic when incrowded or unfamiliar areas^26^. Turano et al. reported that half of the subjects with retinitis pigmentosa experienced falls and 46% had a fear of falling down, these percentages were approximately twice as high as those in a group of age-matches, visually normal subjects^27^. In this study, all of the IMQ scores were lower in patients with HH than in those with monocular blindness. We found that the patients with HH were deeply concerned about their ability to move independently. The homonymous hemianopic visual field defect interfered with patients’ daily living activities related to movement more than monocular blindness.

A previous study reported that younger patients manifested greater difficulty in daily life than older patients, and some studies found that older patients have lower QoL^22,28^. Our study found that patients younger than 55 years of age had significantly more difficulty in outdoor activities than patients aged 55 years and older. When the right and left hemianopia were compared, only the QoL scores for driving were lower in patients with right hemianopia. Drivers in South Korea appear to have more difficulty involving their right rear mirror, because they are seated on the left side. Other questions involving NEI-VFQ and IMQ revealed no significant difference between right and left hemianopia.

Binocular VF test is clinically more relevant because it reflects real binocular visual function. Most models investigating patients with glaucoma indicated a strong correlation between binocular VF and the better eye^29^. Lombardi’s group evaluated the relationships between VF and the performance of patients with glaucoma in simulated daily activities, obtaining better correlations with the MD of the better eye^30^. Burgos-Blasco et al. reported that although the true binocular VF may strongly affect the ability to perform daily activities, it is possible that the QoL perceived by the patient relies more on the MD of the better eye, particularly in large VF defects such as hemianopias^31^. In this study, the MD of the better eye was significantly lower in patients with HH than in the monocular blindness, which can affect the difference in the perceived QoL between the two groups, suggesting that the QoL of HH patients was significantly poorer than that of patients with monocular blindness.

Our study has several limitations. First, the sample size is relatively small. Second, the NEI-VFQ and IMQ are subjective and can be affected by numerous factors, although most studies evaluate QoL based on self-report questionnaires, and it is a widely recognized method. This study is the first of investigating QoL in HH compared with monocular blindness, indicating that MD in better eye affect the daily living.

The results of QoL questionnaires do not represent objectively the functional challenges associated with vision, but reflect their psychological distress, social isolation, depression, behavioral anxiety and fear, and negative influences on their daily life. Visual field loss can impact on a person's ability to participate in rehabilitation, to live in their own home. The QoL survey can be used to evaluate the patient's condition when planning and performing rehabilitation of patients with HH. Patients with HH have lower vision-related QoL and more mobility fears than patients with monocular blindness, which should be strongly considered when treating them. It would be possible to reduce the patients' psychosocial burden after a stroke by providing careful and effective rehabilitation for hemianopia.

## Patients and methods

This prospective, observational survey was designed to compare the QOL of patients with HH and monocular blindness and the association between QOL and VFD. The study protocol adhered to the Declaration of Helsinki and was approved by the Institutional Review Board of Yangsan Pusan National University Hospital (approval no. 05-2020-372). Each study subject provided written informed consent. An independent data and safety monitoring committee provided study oversight. A well-trained coordinator managed the study process.

### Screening

All participants in the study underwent a complete visual function assessment and responded to a self-administered QOL questionnaire. The eye examination included best-corrected visual acuity (BCVA) and a general eye examination identifying pre-existing ocular pathology. The BCVA was measured by a study-certified tester in each eye using the Early Treatment Diabetic Retinopathy Study (ETDRS) protocol. Color perception was tested with Ishihara plates. Patients underwent a Humphrey perimeter 30–2 threshold test with the Swedish Interactive Testing Algorithm (SITA)-Standard program on the Humphrey Visual Field Analyzer (HFA) (Carl Zeiss Meditec, Dublin, CA). The mean deviation (MD) index of the better and worse eyes was recorded.

### Eligibility and exclusion criteria

The major eligibility criteria of the HH group included age between 20 and 80 years (at the time of enrollment), HH resulting from brain lesions, and BCVA of 20/30 or better in each eye. Exclusion criteria were: an ocular etiology for reduced vision, myopia worse than − 6.0 diopters (D) spherical equivalent in any eye, prior intraocular or refractive surgery, diagnosis of glaucoma, and intellectual deficit.

The eligibility criteria of the monocular blindness group were: absence of a history of VFDs and BCVA 20/800 or less in the worse eye. Exclusion criteria were: an ocular co-morbidity that may reduce VA, ocular disease that my reduce visual field, intraocular injury, prior intraocular surgery in better eye, and cognitive delays that may impact testing.

### Protocol

To compare the perceived QOL between the two groups, we administered two questionnaires at baseline. A Korean version of National Eye Institute Visual Function Questionnaire 25 (NEI-VFQ 25) and IMQ were provided to all the enrolled patients. Both questionnaires contained items relating to daily living activities. The patients reported their perceived level of difficulty for each item.

The NEI-VFQ 25 is a global vision-specific health-related QOL questionnaire consisting of 25 items representing 11 subscales and a single item rating general health. The subscales include general vision, ocular pain (2 items), near-vision activities, distance activities, social functioning (4 items), mental health (8 items), role difficulties (5 items), dependency (5 items), driving (4 items), color vision, and peripheral vision. Each of the items includes six responses. The score generated for the VFQ-25 converts the pre-coded numeric values of items to a score ranging from 0 to 100. Higher scores reflect better function and well-being^32^. The interviewer-administered version, translated to Korean, was used^33^.

The IMQ contains items relating to daily living activities. The questionnaire rated the patients’ difficulty in 35 mobility situations. We conducted planned analyses of responses to subsets of questions that we expected would be related to mobility and obstacle avoidance: IMQ questions 1–4, 6–10, 13–17, 24–33, and 35^34^. Subjects were instructed to rate on a scale of 1 to 5 the level of difficulty they experienced in each mobility situation when they did not have an accompanying person or mobility aid to assist them. They were told that 1 meant “no difficulty and 5 denoted “extreme difficulty”; 2, 3, and 4 were not described. They were instructed to score the situation "NA" if they never performed the activity without help. They were told to mark with an "X" any situation that they rated higher than 1 if the difficulty was due to something other than vision loss.

### Statistical analysis

Pearson’s chi-square test was used for categorical variables, and independent Student’s *t* test was used to compare continuous numerical variables. Normality checks were conducted before using the *t* tests. Outcome group comparisons were adjusted for baseline outcome using analysis of covariance. All reported p values were obtained for a two-tailed test. The analyses were conducted using SAS version 9.2 (SAS Institute, Cary, NC, USA).

## Data Availability

The datasets generated during or analysed during the current study are available from the corresponding author on reasonable request.
